# Novel sex-specific influence of parental factors on small-for-gestational-age newborns

**DOI:** 10.1038/s41598-020-76196-x

**Published:** 2020-11-05

**Authors:** Meng Yuan Tian, Shi Wu Wen, Ravi Retnakaran, Hao Ren Wang, Shu Juan Ma, Meng Shi Chen, Xiao Lei Wang, Hui Jun Lin, Hong Zhuan Tan

**Affiliations:** 1 Xiangya School of Public Health, Central South University, Changsha, China; 2 OMNI Research Group, Department of Obstetrics and Gynecology, University of Ottawa, Ottawa, ON Canada; 3 Clinical Epidemiology Program, Ottawa Hospital Research Institute, Ottawa, ON Canada; 4 Leadership Sinai Centre for Diabetes, Mount Sinai Hospital, Toronto, ON Canada; 5 Division of Endocrinology, University of Toronto, Toronto, ON Canada

**Keywords:** Risk factors, Paediatrics, Epidemiology

## Abstract

Since fetal programming is sex-specific, there may also be sex-specific in parental influences on newborn birth weight. We aimed to investigate the influence of parental factors on small-for-gestational-age (SGA) infants of different sexes. Based on a pre-pregnancy cohort, multivariate logistic regression was used. 2275 couples were included for analysis. Significant associations were observed among paternal height, pre-pregnancy body mass index (BMI), and SGA in male infants; among maternal height, pre-pregnancy BMI, and SGA in female infants, and among other maternal factors and SGA in both male and female infants. Such sex specificity may be related to genetic, epigenetic, or hormonal influences between parents and infants. In conclusion, there is a sex specificity in the effect of parental height and pre-pregnancy BMI on SGA. The data suggest that future studies on infants should consider the sex-specific differences between the effects of genetic or environmental factors and infants.

## Introduction

According to the "fetal origin of disease" hypothesis, fetal dysplasia will affect the onset of many chronic diseases, such as cardiovascular disease and diabetes in adults. Therefore, people are paying increasing attention to fetal growth and development and their determinants.

Small-for-gestational age (SGA) infants are defined as those weighing below the 10th percentile of birth weight by sex for a specific completed gestational age in a given reference population^1^. Taking fetal gestational age into account, SGA is considered a more robust indicator of intrauterine fetal development, reflecting the health status of the newborn. Not only can SGA increase the risk of neonatal mortality and morbidity in the perinatal period^2^, but also affect the growth and development of the body and nervous system in childhood^3,4^. In addition, SGA can result in increased risks of chronic diseases, such as cardiovascular disease, obesity, hypertension, and type 2 diabetes, in adulthood^5,6^.

While most studies have confirmed the influence of maternal factors, such as maternal age, education, pre-pregnancy body mass index (BMI), and gestational weight gain, on SGA, the potential contribution of paternal factors to SGA at conception is relatively easy to overlook. In fact, researchers have observed the influence of paternal factors on SGA. Previous studies reported that a lower level of paternal education was associated with the increased risk of SGA^7,8^. Recent findings on the association between paternal age and SGA were inconsistent. Ali et al. found that paternal age between 30 to 45 years was associated with a lower odds of SGA^9^, but another study showed that older paternal age (≥ 40 years) was not associated with the risk of SGA^10^. To our best knowledge, only one study has reported the association between paternal height and SGA. Based on a large birth cohort study including 33,448 pregnant Japanese women, Takagi et al. reported that paternal height was independently associated with SGA infants^10^, suggesting that the effect of paternal height on fetal growth appear to be of genetic. Furthermore, McCowan et al. investigated 2002 couples and observed a relationship between paternal obesity and birth of an SGA infant^11^, but Takagi et al. investigated 33,448 pregnant Japanese women and found that the relationship between paternal BMI and SGA was only significant among male SGA infants^12^, suggesting that paternal BMI may influence the occurrence of SGA in a gender-dependent manner. At present, there are no studies on the influence of paternal factors on SGA in China, while existing studies in western countries or other countries are limited and inconsistent. Thus, further studies with more comprehensive and larger samples in Chinese population are needed.

Previous studies have shown that the sex of the fetus affects maternal and obstetric outcomes. Male sex has been associated with higher incidence of preterm labor, higher cesarean rate, increased risk of cord problems, and increased risk of gestational diabetes mellitus^13–15^. Meanwhile, female sex has been reported to increase the risk of hypertensive diseases of pregnancy and type 2 diabetes mellitus after pregnancy compared with male sex^16,17^. These observed sex differences may be affected by differences in genes and hormones, as well as differences in responses to and interactions with environmental factors^18^. Thus, fetal sex should be taken into consideration while studying adverse pregnancy outcome. Fetal sex may modify the effects of maternal intrauterine environmental changes (nutritional status, stress and exposure to environmental chemicals) on fetal growth and development^19^, and affect early placentation processes^20^. As previous study reported, there is probably a sex-specific maternal-placental-fetal interaction in fetal development^15^. The placental regulations and adaptations are sex dimorphic. The placenta of one sex over the other might possess greater ability to respond and protect against adverse environmental insults^19^. Brown et al. reported that female placental biomarkers were not affected by hyperhomocysteinemia while male placental biomarkers were^20^. Abeelen et al. found that maternal inadequate nutrition in early pregnancy leads to smaller placenta with the decrease in placental size being greater for males than for females^21^. In fact, beginning from conception, the development of male and female embryos can be considered as separate processes because of sex-specific transcriptional regulation. Thus, the fetal programming is sex-specific and fetal growth patterns can be regarded as sex dimorphic. That is, there may be sex differences between male and female fetuses in their responses to environmental factors or fetal genome interaction with environmental factors. There is increasing evidence to support these views. For example, compared to females, male fetuses have a greater sensitivity to maternal BMI and glucose status during pregnancy^22^, as well as more likely to be large for gestational age (LGA) if mothers had high pregestational BMI^23^. There is evidence showed that the effect of multiple micronutrients supplementation during pregnancy appears to have a more significant effect on fetal weight in female fetuses^24^. Meanwhile, Rosa et al. found that increasing prenatal stress associated with increased risk of preterm birth in male infants but not in female infants^25^. In addition, the effect of maternal and paternal height on birth length has been observed to be sex-specific, with paternal height predicting birth length in girls, whereas maternal height predicting birth length in boys^26^. SGA, an adverse pregnancy outcome, is influenced by parental, placental, and fetal factors. Thus, we assumed that such sex-specific differences may also be reflected in the occurrence of SGA.

To date, no study investigated the relationship between parental factors and risk of SGA by sex-stratified analyses. It will be interesting to evaluate whether there is different influence on SGA in different genders of infants. Therefore, based on a pre-pregnancy cohort, we aimed to investigate the association between parental factors and SGA by sex stratification in a relatively large sample of Chinese couples.

## Methods

### Study population

**Figure 1:**
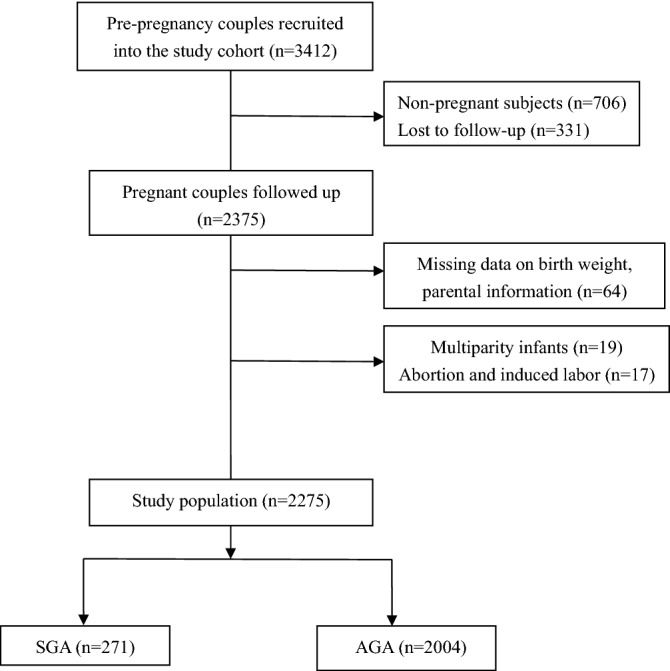
The flow chart of sample screening.

The study population was selected from a pre-conception birth cohort, with 3412 couples recruited at the time of a pre-pregnancy examination in the city of Liuyang in Hunan Province in China between February 2009 and July 2017. The exclusion criteria were as follows: (1) twins and other multiple births, (2) aborted pregnancies or induced labor, (3) no birth weight information, or (4) more than 20% of parental information was missing. Finally, 2275 couples were included for analysis (Fig. 1). The study has been approved by Committee for Human Research Protections, Xiang-Ya School of Medicine, Central South University, and all methods were performed in accordance with relevant guidelines and regulations, and all participants provided written informed consent.

### Recruitment and data collection

We chose to establish a pre-pregnancy cohort at Liuyang Maternal and Infant Hospital in Hunan province, which recruited pre-pregnancy couples who planned to become pregnant in the next 6 months. At the time of enrollment, baseline data of pre-pregnancy couples such as socioeconomic characteristics, living habits, and previous medical history were obtained through questionnaires. Their height, weight, waist circumference, and blood pressure (BP) were measured by a trained nurse. Using a standard height and weight scale (Jiangsu Suhong Medical Devices Co., Ltd, China), parental height was measured to the nearest 0.1 cm, and weight was measured to the nearest 0.1 kg, with light clothing without shoes. We then followed-up pregnant women from pregnancy up to delivery, gathering pregnancy-related information such as pregnancy complications, biochemical variables, weight, waist circumference, and BP through their prenatal health care manual. After delivery, data of gestational age, infant sex, and birth weight were collected from the electronic medical record system of the hospital.

### Variable definitions and diagnostic criteria

According to the birth weight standards for Chinese newborns of different gestational ages formulated by the China Neonatal Network in 2015^27^, if a newborn’s birth weight is lower than the 10th percentile of the average weight of newborns of the same sex and gestational age, they are defined as SGA; whereas if a newborn’s birth weight is higher than the 90th percentile of the average weight of newborns of the same sex and gestational age, they are defined as LGA. Appropriate for gestational age (AGA) is defined as a newborn’s birth weight between the 10th and 90th percentiles of the average weight of newborns of the same sex and gestational age. Subjects were stratified according to pre-pregnancy BMI (kg/m^2^): underweight (BMI < 18.5), normal weight (18.5 ≤ BMI < 24.0), overweight (24.0 ≤ BMI < 28.0), or obesity (BMI ≥ 28.0), according to the standards for Chinese adults proposed by the Chinese Working Group on Obesity^28^. According to the Institute of Medicine recommendation in 2009^29^, the weight gain target during pregnancy was 12.5–18.0 kg for women with underweight, 11.5–16.0 kg for women with normal weight, 7–11.5 kg for women with overweight, and 5–9 kg for women with obesity. Weight gain during pregnancy was classified into three groups: inadequate, adequate, and excessive. Moreover, according to the median height and weight of the parents, paternal height (P_50_ = 169.0; < 169 cm, and ≥ 169 cm), maternal height (P_50_ = 158.0; < 158 cm, ≥ 158 cm), paternal weight (P_50_ = 63.0; < 63 kg, ≥ 63 kg), and maternal weight (P_50_ = 50.0; < 50 kg, ≥ 50 kg) were divided into two groups. Gestational hypertension was defined by systolic BP ≥ 140 mmHg and/or diastolic BP ≥ 90 mmHg at 20 gestational weeks, based on the average of at least two measurements. The diagnosis of gestational diabetes mellitus was based on the diagnostic criteria established by the American Diabetes Association in 2011: fasting glucose ≥ 5.1 mmol/L or glucose ≥ 10.0 mmol/L for 1 h after oral glucose loading (75 g) or glucose ≥ 8.5 mmol/L for 2 h.

### Statistical analysis

Epidata 3.1 software (Epidata Association; Odense, Denmark) was used to input information, Microsoft Excel 2010 (Microsoft, Redmond, WA) was used to sort the data, and SPSS 22.0 software (IBM; Armonk, NY) was used to establish the database and conduct statistical analysis. The types of data collected included quantitative, qualitative, and ranked data. For descriptions of data, mean (± SD) is used for quantitative data and No. (%) for qualitative and ranked data. To study the influence of each factor on SGA in both infant sexes, we conducted univariate and multivariate analyses by sex stratification. In univariable analysis, the independent sample *t*-test was used to compare means, and an R × C contingency table test was used for rate comparison. Then, to control for potential confounders, stepwise multivariate logistic regression analysis (α_enter_ = 0.05, α_out_ = 0.10) was performed. The dependent variable was SGA; the independent variables were those that showed significant associations with SGA in univariate analysis; and the covariables included maternal age, maternal passive smoking before pregnancy, and parity. Because weight is closely associated with BMI, there may be collinearity in regression analysis, and as BMI better predicts obesity, we selected BMI as the independent variable in regression analysis. In order to adjust the possible bias from LGA, we carried out another multivariable regression analysis after excluded LGA. A *P* value < 0.05 was considered to be statistically significant (*P* values are 2 sided).

## Results

### Sample characteristics

A total of 2275 couples and newborns were included in our database. The prevalence of SGA was 11.91% (271/2275). In the SGA infants, 164 were male (60.52%) and 107 were female (39.48%). The mean age, average height, and average weight were 24.0 years, 157.6 cm, and 49.6 kg in mothers, 26.0 years, 168.9 cm, and 62.7 kg in father, respectively. Detailed information is shown in Table 1.

**Table 1:** Description of parental main characteristics.

Characteristics	Mother	Father
**Age, years, no. (%)**		
≤ 24	1418 (62.3)	753 (33.1)
25–29	743 (32.7)	1256 (55.2)
≥ 30	114 (5.0)	266 (11.7)
**Education, years, no. (%)**		
≤ 9	986 (43.3)	989 (43.5)
10–	951 (41.8)	962 (42.3)
≥ 13	338 (14.9)	324 (14.2)
**Average annual household income, CNY, No. (%)**		
< 50,000	1245 (54.7)	1245 (54.7)
≥ 50,000	1030 (45.3)	1030 (45.3)
Height, mean (± SD), cm	157.62 (± 4.20)	168.93 (± 3.88)
Weight before pregnancy, mean (± SD), kg	49.86 (± 5.61)	62.85 (± 6.72)
**BMI before pregnancy, no. (%)**		
Underweight	443 (19.5)	119 (5.2)
Normal	1713 (75.3)	1859 (81.7)
Overweight/obese	119 (5.2)	297 (13.1)
**Central obesity before pregnancy, no. (%)**		
Yes	165 (7.3)	215 (9.5)
No	2110 (92.7)	2060 (90.5)
**Smoking before pregnancy, no. (%)**		
Yes	8 (0.4)	1061 (59.2)
No	2029 (99.6)	732 (40.8)
**Passive smoking before pregnancy, no. (%)**		
Yes	96 (4.2)	110 (10.8)
No	2179 (95.8)	906 (89.2)

*BMI* body mass index, *SD* standard deviation.

### Univariate analysis of SGA determinants according to fetus sex

For total subjects, we found that paternal height, weight, BMI, maternal height, weight, BMI, gestational hypertension, oligohydramnios, and weight gain during pregnancy were significantly different between SGA and AGA (appropriate for gestational age, AGA) group (*P* < 0.05) (Tables 2 and 3). By gender stratification, there were significant differences in paternal height, and BMI between male SGA and AGA infants (*P* < 0.05) (Table 2). Maternal height, and BMI were found significantly different between female SGA and AGA infants. Parental weight, weight gain during pregnancy, gestational hypertension, and oligohydramnios had effects on both male and female SGA infants (*P* < 0.05) (Table 3). Paternal age, education, smoking history, average annual household income, maternal education, and gestational diabetes were not significantly different from all SGA infants (*P* > 0.05).

**Table 2:** Univariate analysis of the association between paternal factors and SGA by sex stratification.

Variables	All (n = 2275)			Male (n = 1193)			Female (n = 1082)		
	SGA	AGA	*P*	SGA	AGA	*P*	SGA	AGA	*P*
**Paternal age, no. (%)**									
≤ 24	93 (12.4)	660 (87.6)	0.731	56 (14.4)	333 (85.6)	0.740	37 (10.2)	327 (89.8)	0.206
25–29	150 (11.9)	1106 (88.1)		87 (13.1)	578 (86.9)		63 (10.7)	528 (89.3)	
≥ 30	28 (10.5)	238 (89.5)		21 (15.1)	118 (84.9)		7 (5.5)	120 (94.5)	
**Paternal education, no. (%)**									
≤ 9	126 (12.7)	863 (87.3)	0.309	85 (16.7)	425 (83.3)	0.034	41 (8.6)	438 (91.4)	0.255
10–	114 (11.9)	848 (88.1)		61 (12.0)	446 (88.0)		53 (11.6)	402 (88.4)	
≥ 13	31 (9.6)	293 (90.4)		18 (10.2)	158 (89.8)		13 (8.8)	135 (91.2)	
**Average annual household income, no. (%)**									
< 50,000	159 (12.8)	1086 (87.2)	0.164	100 (15.1)	563 (84.9)	0.134	59 (10.1)	523 (89.9)	0.768
≥ 50,000	112 (10.9)	918 (89.1)		64 (12.1)	466 (87.9)		48 (9.6)	452 (90.4)	
**Paternal smoking before pregnancy, no. (%)**									
Yes	123 (11.6)	938 (88.4)	0.730	75 (13.2)	493 (86.8)	0.646	48 (9.7)	445 (90.3)	0.292
No	81 (11.1)	651 (88.9)		54 (14.2)	325 (85.8)		27 (7.6)	326 (92.4)	
**Paternal passive smoking before pregnancy, no. (%)**									
Yes	16 (14.5)	94 (85.5)	0.275	10 (20.4)	39 (79.6)	0.294	6 (9.8)	55 (90.2)	0.441
No	100 (11.0)	806 (89.0)		69 (14.7)	399 (85.3)		31 (7.1)	407 (92.9)	
**Paternal height group, no. (%)**									
< 169 cm	150 (14.0)	924 (86.0)	0.004	93 (16.7)	465 (83.3)	0.006	57 (11.0)	459 (89.0)	0.223
≥ 169 cm	121 (10.1)	1080 (89.9)		71 (11.2)	564 (88.8)		50 (8.8)	516 (91.2)	
**Paternal weight group, no. (%)**									
< 63 kg	174 (13.6)	1105 (86.4)	0.005	102 (15.6)	551 (84.4)	0.039	72 (11.5)	554 (88.5)	0.037
≥ 63 kg	97 (9.7)	899 (90.3)		62 (11.5)	478 (88.5)		35 (7.7)	421 (92.3)	
**Paternal BMI before pregnancy, no. (%)**									
Underweight	24 (20.2)	95 (79.8)	0.001	18 (30.0)	42 (70.0)	0.000	6 (10.2)	53 (89.8)	0.512
Normal	225 (12.1)	1634 (87.9)		135 (13.7)	852 (86.3)		90 (10.3)	782 (89.7)	
Overweight/obese	22 (7.4)	275 (92.6)		11 (7.5)	135 (92.5)		11 (7.3)	140 (92.7)	

*BMI* body mass index.

**Table 3:** Univariate analysis of the association between maternal factors and SGA by sex stratification.

Variables	All (n = 2275)			Male (n = 1193)			Female (n = 1082)		
	SGA	AGA	*P*	SGA	AGA	*P*	SGA	AGA	*P*
**Maternal age, no. (%)**									
≤ 24	189 (13.3)	1229 (86.7)	0.017	116 (15.2)	645 (84.8)	0.013	73 (11.1)	584 (88.9)	0.168
25–29	68 (9.2)	675 (90.8)		37 (9.8)	342 (90.2)		31 (8.5)	333 (91.5)	
≥ 30	14 (12.3)	100 (87.7)		11 (20.8)	42 (79.2)		3 (4.9)	58 (95.1)	
**Maternal education, no. (%)**									
≤ 9	116 (11.8)	870 (88.2)	0.322	67 (13.4)	434 (86.6)	0.812	49 (10.1)	436 (89.9)	0.245
10–	122 (12.8)	829 (87.2)		74 (14.5)	438 (85.5)		48 (10.9)	391 (89.1)	
≥ 13	33 (9.8)	305 (90.2)		23 (12.8)	157 (87.2)		10 (6.3)	148 (93.7)	
**Maternal height, no. (%)**									
< 158 cm	160 (14.1)	972 (85.9)	0.001	87 (15.0)	494 (85.0)	0.230	73 (13.4)	473 (86.6)	0.000
≥ 158 cm	111 (9.7)	1032 (90.3)		77 (12.6)	535 (87.4)		34 (6.3)	502 (93.7)	
**Maternal weight, no. (%)**									
< 50 kg	179 (15.1)	1007 (84.9)	0.000	105 (17.0)	511 (83.0)	0.001	74 (13.0)	496 (87.0)	0.000
≥ 50 kg	92 (8.4)	997 (91.6)		59 (10.2)	518 (89.8)		33 (6.4)	479 (93.6)	
**Maternal BMI before pregnancy, no. (%)**									
Underweight	71 (16.0)	372 (84.0)	0.008	36 (16.5)	182 (83.5)	0.340	35 (15.6)	190 (84.4)	0.005
Normal	190 (11.1)	1532 (88.9)		121 (13.3)	787 (86.7)		69 (8.6)	736 (91.4)	
Overweight/obese	10 (8.4)	109 (91.6)		7 (10.4)	60 (89.6)		3 (5.8)	49 (94.2)	
**Maternal smoking before pregnancy, no. (%)**									
Yes	1 (12.5)	7 (87.5)	0.921	1 (20.0)	4 (80.0)	0.666	0 (0.0)	3 (100.0)	0.584
No	231 (11.4)	1798 (88.6)		145 (13.4)	937 (86.6)		86 (9.1)	861 (90.9)	
**Maternal passive smoking before pregnancy, no. (%)**									
Yes	19 (19.8)	1927 (88.4)	0.015	13 (25.0)	39 (75.0)	0.016	6 (13.6)	38 (86.4)	0.395
No	252 (11.6)	77 (80.2)		151 (13.2)	990 (86.8)		101 (9.7)	937 (90.3)	
**Parity, no. (%)**									
≤ 1	233 (12.3)	1664 (87.7)	0.014	141 (14.1)	858 (85.9)	0.154	92 (10.2)	806 (89.8)	0.035
> 1	25 (7.6)	305 (92.4)		17 (10.1)	152 (89.9)		8 (5.0)	153 (95.0)	
**Gestational diabetes, no. (%)**									
Yes	4 (7.1)	52 (92.9)	0.265	4 (12.1)	29 (87.9)	0.783	0 (0.0)	23 (100.0)	0.108
No	267 (12.0)	1952 (88.0)		160 (13.8)	1000 (86.2)		107 (10.1)	952 (89.9)	
**Gestational hypertension, no. (%)**									
Yes	24 (29.3)	58 (70.7)	0.000	16 (32.7)	33 (67.3)	0.000	8 (24.2)	25 (75.8)	0.005
No	247 (11.3)	1946 (88.7)		148 (12.9)	996 (87.1)		99 (9.4)	950 (90.6)	
**Oligohydramnios, no. (%)**									
Yes	31 (21.7)	112 (78.3)	0.000	17 (24.6)	52 (75.4)	0.007	14 (18.9)	60 (81.1)	0.007
No	240 (11.3)	1892 (88.7)		147 (13.1)	977 (86.9)		93 (9.2)	915 (90.8)	
**Weight gain during pregnancy, no. (%)**									
Inadequate	57 (16.2)	294 (83.8)	0.000	31 (16.7)	155 (83.3)	0.012	26 (15.8)	139 (84.2)	0.000
Adequate	197 (12.5)	1375 (87.5)		120 (14.6)	702 (85.4)		77 (10.3)	673 (89.7)	
Excessive	17 (4.8)	335 (95.2)		13 (7.0)	172 (93.0)		4 (2.4)	163 (97.6)	

*BMI* body mass index.

### Multivariate analysis of SGA determinants according to fetus sex

The assignment of variables included in the multivariate logistic regression analysis is shown in Table 4. Multivariate analysis showed that maternal height (shorter height odds ratio (OR) 2.36, 95% confidence interval (CI) 1.50–3.72) and maternal BMI (underweight OR 1.91, 95% CI 1.19–3.06) were significantly associated with SGA in female infants only. Paternal height (shorter height OR 1.52, 95% CI 1.07–2.15) and BMI (overweight/obesity OR 0.48, 95% CI 0.25–0.93; underweight OR 2.92, 95% CI 1.59–5.36) were significantly associated with SGA in male infants only. Weight gain during pregnancy (for male, OR 0.35, 95% CI 0.19–0.65; for female, OR 0.23, 95% CI 0.08–0.67), gestational hypertension (for male, OR 3.92, 95% CI 2.03–7.55; for female, OR 3.74, 95% CI 1.53–9.13), and oligohydramnios (for male, OR 2.49, 95% CI 1.37–4.52; for female, OR 2.40, 95% CI 1.24–4.64) were associated with SGA in both male and female infants. The result of regression analysis after excluded LGA was almost the same as before (Table 5).

**Table 4:** Variables included in the multivariate regression analysis assignment.

Variable	Assignment
BMI	1 = “normal”, 2 = “underweight”, 3 = “overweight/obese”, “normal” as the reference
Age	1 = “ ≤ 24”, 2 = “25–”, 3 = “ ≥ 30”, “ ≤ 24”as the reference
Maternal passive smoking before pregnancy	0 = “no”,1 = “yes”
Educational years	1 = “ ≤ 9”, 2 = “10-”, 3 = “ ≥ 13” “ ≤ 9”as the reference
Parity	1 = “ ≤ 1”, 2 = “ > 1”
Oligohydramnios	0 = “no”, 1 = “yes”
Gestational hypertension	0 = “no”, 1 = “yes”
Weight gain during pregnancy	1 = “Adequate”, 2 = “Inadequate”, 3 = “Excessive”, “adequate” as the reference
Paternal height	1 = “ ≥ 169 cm”, 2 = “ < 169 cm”, “1 = ≥ 169 cm” as the reference
Maternal height	1 = “ ≥ 158 cm”, 2 = “ < 158 cm”, “1 = ≥ 158 cm” as the reference
SGA	0 = “no”, 1 = “yes”

*BMI* body mass index, *SGA* small for gestational age.

**Table 5:** Multivariate analysis of the association between parental factors and SGA by different gender fetal.

	Control group included LGA			Control group excluded LGA		
	β	OR	95% CI	β	OR	95% CI
**Male infants**						
Paternal BMI						
Normal	Reference			Reference		
Underweight	1.07	2.92	1.59, 5.36	0.98	2.67	1.43, 4.98
Overweight/obese	− 0.73	0.48	0.25, 0.93	− 0.75	0.48	0.25, 0.92
Paternal height	0.42	1.52	1.07, 2.15	0.40	1.50	1.04, 2.14
Weight gain during pregnancy						
Adequate	Reference			Reference		
Inadequate	0.09	1.10	0.70, 1.71	0.06	1.06	0.68, 1.67
Excessive	− 1.05	0.35	0.19, 0.65	− 1.01	0.37	0.20, 0.68
Oligohydramnios	0.91	2.49	1.37, 4.52	0.88	2.41	1.32, 4.41
Gestational hypertension	1.37	3.92	2.03, 7.55	1.59	4.90	2.47, 9.75
**Female infants**						
Maternal BMI before pregnancy						
Normal	Reference			Reference		
Underweight	0.65	1.91	1.19, 3.06	0.70	2.01	1.27, 3.18
Overweight/obese	0.54	1.72	0.46, 6.42	0.55	1.73	0.46, 6.52
Maternal height	0.86	2.36	1.50, 3.72	0.85	2.34	1.49, 3.68
Weight gain during pregnancy						
Adequate	Reference			Reference		
Inadequate	0.30	1.36	0.80, 2.31	0.37	1.45	0.88, 2.39
Excessive	− 1.47	0.23	0.08, 0.67	− 1.53	0.22	0.07, 0.64
Oligohydramnios	0.87	2.40	1.24, 4.64	0.83	2.29	1.19, 4.43
Gestational hypertension	1.32	3.74	1.53, 9.13	1.30	3.67	1.50, 8.98

*BMI* body mass index, *OR* odds ratio, *CI* confidence interval.

## Discussion

In our study, we used a pre-birth cohort with a relatively large sample size to examine the association between parental factors and SGA by sex stratification. The results showed that the association among paternal height, BMI, and SGA is significant only in male infants, the association among maternal height, BMI, and SGA is significant only in female infants, and the associations between other maternal factors and SGA is significant in both male and female infants. Our findings are important for understanding the role of parental factors and providing clues of studying the possible genetic mechanism in the occurrence of SGA.

We found sex specificity for the effects of parental height on SGA. Parental height is known to be important factor associated with the fetal growth of offspring. Recent studies observed a significantly association between maternal short stature and the increased risk of SGA^30,31^. Shorter mothers may have pelvic stenosis and smaller uterine volumes, resulting in limited intrauterine fetal growth^32^. They may also suffer from chronic malnutrition, leading to inadequate supply of nutrients to their fetuses^32^, and be more susceptible to infections during pregnancy^33^. These factors, combined with genetic factors, may contribute to the development of SGA. There is some evidence that paternal height is an independent predictor of offspring birth weight^34,35^, but these studies were not specified for SGA. The related study about the effect of paternal height on SGA is rare. As far as we know, only one study, a large birth cohort study (JECS) in Japan, reported that paternal height was associated with the odds of SGA in both male and female infants^12^. A previous study demonstrate that fetal birth size appears to be, in part, heritable through the paternal germ line^36^, suggesting that the influence of paternal height on birth weight or SGA may be genetic. Paternal height may influence the growth and development of the offspring by passing on the DNA sequence of germ cells, furthermore, the epigenetic changes of germ cells due to the father's lifestyle and nutrition changes, affecting the growth of the fetus.

Maternal BMI reflects the intrauterine environment of fetal growth, and may have a direct physiological impact on fetal growth through factors such as nutrient supply and hormone distribution^37^. Our results not only showed that maternal underweight is associated with an increased risk of SGA, but also innovatively found that this association is significant only in female infants. Several studies have reported that maternal pre-pregnancy underweight increases the risk of SGA^38,39^, but not specified for sex. However, a Spanish prospective study investigated 9270 pregnant women and found a significantly negative association between maternal BMI and male SGA infants^23^, which is inconsistent with our result. On the one hand, it may be related to racial differences. Genetic or genetic phenotypic characteristics may vary by ethnicity. On the other hand, it may be due to differences in controlled confounders. Our study controlled for potential confounders such as maternal height, maternal age, parity, history of passive smoking, weight gain during pregnancy, oligohydramnios, and pregnancy-induced hypertension, while this Spanish study controlled only for maternal age, gestational age at delivery, glucose tolerance, and pregnancy-induced hypertension. Some evidence from animal experiments and human studies have indicated an important biological role of paternal weight in offspring birth weight^40,41^. Earlier study reported that paternal obesity is associated with SGA^11^, but recent studies have found that paternal BMI affects growth of the male^42^, and was significantly associated with SGA only in male infants^12^, which is consistent with our results, suggesting that paternal BMI may affect SGA in a gender-dependent manner. Epigenetic mechanisms might play an important role in the influence of paternal environment on fetal growth. There is evidence that paternal environmental information is transmitted to offspring via sperm and that small RNAs are environmentally responsive epigenetic molecules in sperm. Remodeling of sperm small RNA payload may have consequences for early embryonic development and offspring health^43^.

Although the underlying mechanisms of the sex-specific influences of parental height and BMI on SGA are unclear, it is possible that genetic and environmental regulators of fetal growth are affected by fetal sex^44^. For instance, Lampl and colleagues reported that sex modified the effects of maternal height and weight on fetal growth rates and birth weight^45^. We try to explain it from the following aspects. Firstly, in the early days, gene expression by the sex chromosomes was unequal between males and females^18^. This difference results in sex-specific differential expression of sex chromosome encoded genes and affects the transcription of autosomal genes^46^. One study concluded that sex-specific male-line transgenerational responses exist in humans and hypothesized that these transmissions are mediated by the sex chromosomes^47^. Thus, males with Y chromosome may be more sensitive to paternal effects. Secondly, during pregnancy, differences in gene expression may lead to placental functional differences, leading to sex-specific alterations in fetal development^48^. The placenta, as an interface between the mother and fetus, is a key moderator of fetal growth and development, which plays a key role in buffering environmental effects transmitted by the mother^46^. Previous study indicated that the consequences of maternal exposure to metabolism, diet and hormonal changes are transmitted from the maternal to the fetal compartment via placenta in a sex-specific manner and affects fetal development^15^. Recent data also indicated that paternal condition changes affect placenta alterations, which are also sex specific^19^. Thus, due to placental sexually dimorphic, regardless of the genetic or environment factor effect, both maternal and paternal height and weight have impacts on the growth and development of children, fetal responses to these effects are sex specific. That is, male and female fetuses may establish different strategies to cope with the same adverse intrauterine environment^48,49^. Maternal short stature or pre-pregnancy underweight are likely to provide an adverse intrauterine nutritional environment. In this case, we assume that female fetuses may be more sensitive than male fetuses, and easy to develop to SGA. Thirdly, male and female fetuses have differential responses to the same environment via epigenetic mechanisms^50^. Environmental factors such as nutrients or compounds can affect epigenetic markers in a sex-dependent manner during specific developmental periods of intrauterine life^42^. Studies have shown that sex specific epigenetic patterns changes are associated with birth weight^51,52^. Thus, fetal sex-specific epigenetic patterns may provide a possible explanation for the sex-specific effect of parental height and BMI on SGA infants. Obviously, the underlying mechanisms should be further explored.

Our findings have some significance. First, we should not only pay attention to the optimization of maternal health before pregnancy but also the health status of the father before pregnancy, so as to achieve transgenerational benefits. Second, it is suggested that future studies on newborns should consider the sex-specific differences between the effects of genetic or environmental factors and infants.

This study has some limitations. First, pre-pregnancy passive smoking was self-reported and may lead to some misclassification of actual exposure status. The lack of more information collection on factors influencing SGA, such as dietary nutrition, physical activity and psychological status of pregnant women during pregnancy. These unadjusted potential confounding factors may influence the results of this study. Second, the pre-pregnancy weight collected within 6 months may be different from the exact pre-pregnancy weight. We compared the pre-pregnancy weight within 6 months and the weight in early pregnancy (gestational age < 10 weeks, which may be very close to the exact pre-pregnancy weight) and found no significant difference between the 2 time weight, which may indicating the possible bias caused by weight change within six months could be ignored (detailed analysis results provided in supplement material). Third, we have not further investigated the possible mechanism of our finding. Finally, the participants of this study comprised a single ethnicity (Chinese), and whether the research results can be extended to other ethnicity population still needs further confirmation.

## Conclusion

In conclusion, we identified a sex specificity in the effect of parental height and BMI on SGA after controlling for potential confounding factors. That is, SGA was associated with paternal height and BMI in male infants; while in female infants, SGA was associated with maternal height and BMI. Such sex specificity may be related to genetic, epigenetic, or hormonal influences between parents and infants. Obviously, the underlying mechanisms need to be further explored in molecular genetics or epigenetics. It will be clinical interest to reveal the role of the sex specificity of the fetus to better understand the impact of parental risk factors on SGA, and to improve the early diagnosis, prevention and treatment of SGA.

## Supplementary information

Supplementary Tables.
