# Supplementary material for: Novel sex-specific influence of parental factors on small-for-gestational-age newborns
Source: Sci Rep. 2020 Nov 5;10:19226. doi: 10.1038/s41598-020-76196-x (PMC7644766; doi:10.1038/s41598-020-76196-x)
Supplement: Supplementary file 1 — Supplementary Tables. [file 41598_2020_76196_MOESM1_ESM.docx]

**Supplementary material**

**Novel sex-specific influence of parental factors on small-for-gestational-age newborns**

Meng-yuan Tian^1^; ShiWuWen^2,3^; Ravi Retnakaran^4,5^; Hao-ren Wang^1^; Shu-juan Ma^1^; Meng-shi Chen^1^; Xiao-lei Wang^1^; Hui-jun Lin^1^; Hong-zhuan Tan^1^*

1. Xiangya School of Public Health, Central South University, Changsha, China

2. OMNI Research Group, Department of Obstetrics and Gynecology, University of Ottawa, Ottawa, Ontario, Canada

3. Ottawa Hospital Research Institute, Clinical Epidemiology Program, Ottawa, Ontario, Canada

4. Leadership Sinai Centre for Diabetes, Mount Sinai Hospital, Toronto, Ontario, Canada

5. Division of Endocrinology, University of Toronto, Toronto, Ontario, Canada

*E-mail: tanhz99@qq.com

Supplementary Table 1. Paired sample statistics

|  | Mean value | N | Standard deviation | Standard error mean |
| --- | --- | --- | --- | --- |
| Pre-pregnancy weight | 49.2012 | 63 | 6.09017 | 0.76729 |
| Weight in early pregnancy | 49.6696 | 63 | 6.58366 | 0.82946 |

Supplementary Table 2. Paired sample test

|  | Mean value | Standard deviation | Standard error mean | 95% Confidence Interval | T | P |
| --- | --- | --- | --- | --- | --- | --- |
| Pre-pregnancy weight vs. Weight in early pregnancy | 0.46842 | 3.99549 | 0.50338 | -0.53783,1.47468 | 0.931 | 0.356 |

Supplementary Table 3. Univariate analysis of the association between parental anthropometric indicators and SGA based on a national reference standard in China

| Variables | All (n=2275) | | | Male (n= 1193) | | | Female (n=1082) | | | |
| --- | --- | --- | --- | --- | --- | --- | --- | --- | --- | --- |
|  | SGA | AGA | *P* | SGA | AGA | *P* | | SGA | AGA | *P* |
| Paternal height group,  No. (%) |  |  |  |  |  |  | |  |  |  |
| ＜168.8 cm | 114(13.7) | 716(863) | 0.042 | 75(17.0) | 367(83.0) | 0.013 | | 39(10.1) | 349(89.9) | 0.894 |
| ≥168.8 cm | 157(10.9) | 1288(89.1) |  | 89(11.9) | 662(88.1) |  | | 68(9.8) | 626(90.2) |  |
| Paternal weight group, No. (%) |  |  |  |  |  |  | |  |  |  |
| ＜66.2 kg | 228(12.9) | 1535(7.1) | 0.005 | 137(14.8) | 786(85.2) | 0.042 | | 91(10.8) | 749 (89.2) | 0.053 |
| ≥66.2 kg | 43(8.4) | 469(91.6) |  | 27(10.0) | 243(90.0) |  | | 16(6.6) | 226(93.4) |  |
| Maternal height group, No. (%) |  |  |  |  |  |  | |  |  |  |
| ＜158.6 cm  ≥158.6 cm | 197(13.7)  74(8.9) | 1243(86.3)  761(91.1) | 0.001 | 112(14.9)  52(11.7) | 638(85.1)  391(88.3) | 0.121 | | 85(12.3)  22(5.6) | 605(87.7)  370(94.4) | 0.000 |
| Maternal weight group, No. (%) |  |  |  |  |  |  | |  |  |  |
| ＜57.3 kg  ≥57.3 kg | 253(12.4)  18(7.5) | 1783(87.6)  221(92.5) | 0.027 | 151(14.3)  13(9.7) | 908(85.7)  121(90.3) | 0.149 | | 102(10.4)  5(4.8) | 875(89.6)  100(95.2) | 0.064 |

Supplementary Table 4. Multivariate analysis of the association between parental anthropometric indicators and SGA based on a national reference standard in China

|  | Control group included LGA | | |  | Control group excluded LGA | | |
| --- | --- | --- | --- | --- | --- | --- | --- |
|  | β | OR | 95% CI |  | β | OR | 95% CI |
| Male infants |  |  |  |  |  |  |  |
| Paternal BMI |  |  |  |  |  |  |  |
| Normal | Reference |  |  |  | Reference |  |  |
| Underweight | 1.15 | 3.15 | 1.69,5.87 |  | 0.16 | 1.17 | 0.74,1.85 |
| Overweight/Obese | -0.743 | 0.48 | 0.25,0.92 |  | -0.97 | 0.38 | 0.20,0.71 |
| Paternal height | 0.42 | 1.53 | 1.06,2.19 |  | 0.38 | 1.46 | 1.01,2.11 |
| Weight gain during pregnancy |  |  |  |  |  |  |  |
| Adequate | Reference |  |  |  | Reference |  |  |
| Inadequate | 0.19 | 1.21 | 0.77,1.91 |  | 1.10 | 3.00 | 1.58,5.69 |
| Excessive | -0.98 | 0.38 | 0.20,0.70 |  | -0.78 | 0.46 | 0.23,0.89 |
| Oligohydramnios | 0.88 | 2.42 | 1.33,4.41 |  | 0.86 | 2.36 | 1.28,4.32 |
| [Gestational hypertension](http://www.youdao.com/w/gestational%20hypertension/#keyfrom=E2Ctranslation) | 1.48 | 4.40 | 2.27,8.52 |  | 1.72 | 5.56 | 2.77,11.14 |
| Female infants |  |  |  |  |  |  |  |
| Maternal BMI before pregnancy |  |  |  |  |  |  |  |
| Normal | Reference |  |  |  | Reference |  |  |
| Underweight | 0.64 | 1.89 | 1.18,3.03 |  | 0.68 | 1.97 | 1.23,3.17 |
| Overweight/Obese | 0.56 | 1.75 | 0.47,6.59 |  | 0.62 | 1.85 | 0.48,7.08 |
| Maternal height | 1.02 | 2.78 | 1.64,4.71 |  | 1.00 | 2.73 | 1.61,4.64 |
| Weight gain during pregnancy |  |  |  |  |  |  |  |
| Adequate | Reference |  |  |  | Reference |  |  |
| Inadequate | 0.38 | 1.46 | 0.85,2.49 |  | 0.31 | 1.36 | 0.80,2.33 |
| Excessive | -1.51 | 0.22 | 0.08,0.65 |  | -1.54 | 0.21 | 0.07,0.64 |
| Oligohydramnios | 0.93 | 2.53 | 1.31,4.91 |  | 0.93 | 2.54 | 1.31,4.93 |
| [Gestational hypertension](http://www.youdao.com/w/gestational%20hypertension/#keyfrom=E2Ctranslation) | 1.34 | 3.82 | 1.56,9,35 |  | 1.38 | 3.98 | 1.62,9.78 |

Abbreviation: BMI, body mass index; OR, odds ratio; CI, confidence interval
